# Fatty acid composition of developing tree peony (*Paeonia* section *Moutan* DC.) seeds and transcriptome analysis during seed development

**DOI:** 10.1186/s12864-015-1429-0

**Published:** 2015-03-18

**Authors:** Shan-Shan Li, Liang-Sheng Wang, Qing-Yan Shu, Jie Wu, Li-Guang Chen, Shuai Shao, Dan-Dan Yin

**Affiliations:** Key Laboratory of Plant Resources and Beijing Botanical Garden, Institute of Botany, Chinese Academy of Sciences, Beijing, 100093 China; University of Chinese Academy of Sciences, Beijing, 100049 China; Institute of Forest Protection, Chinese Academy of Forestry, Beijing, 100091 China; College of Horticulture, Nanjing Agricultural University, Nanjing, 210095 China

**Keywords:** *Paeoniaceae*, Tree peony, Transcriptome, α-linolenic acid, Omega-3 fatty acid, Triacylglycerols

## Abstract

**Background:**

Tree peony (*Paeonia* section *Moutan* DC.) is known for its excellent ornamental and medicinal values. In 2011, seeds from *P. ostii* have been identified as novel resource of α-linolenic acid (ALA) for seed oil production and development in China. However, the molecular mechanism on biosynthesis of unsaturated fatty acids in tree peony seeds remains unknown. Therefore, transcriptome data is needed to better understand the underlying mechanisms.

**Results:**

In this study, lipid accumulation contents were measured using GC-MS methods across developing tree peony seeds, which exhibited an extraordinary ALA content (49.3%) in *P. ostii* mature seeds. Transcriptome analysis was performed using Illumina sequencing platform. A total of 144 million 100-bp paired-end reads were generated from six libraries, which identified 175,874 contigs. In the KEGG Orthology enrichment of differentially expressed genes, lipid metabolism pathways were highly represented categories. Using this data we identified 388 unigenes that may be involved in *de novo* fatty acid and triacylglycerol biosynthesis. In particular, three unigenes (*SAD*, *FAD2* and *FAD8*) encoding fatty acid desaturase with high expression levels in the fast oil accumulation stage compared with the initial stage of seed development were identified.

**Conclusions:**

This study provides the first comprehensive genomic resources characterizing tree peony seeds gene expression at the transcriptional level. These data lay the foundation for further understanding of molecular mechanism responsible for lipid biosynthesis and the high unsaturated fatty acids (especially ALA) accumulation. Meanwhile, it provides theoretical base for potential oilseed application in the respect of n-6 to n-3 ratio for human diets and future regulation of target healthy components of oils.

**Electronic supplementary material:**

The online version of this article (doi:10.1186/s12864-015-1429-0) contains supplementary material, which is available to authorized users.

## Background

Tree peony (*Paeonia* section *Moutan* DC.) is a perennial deciduous shrub with excellent ornamental and medicinal values [1-3]. It is indigenous to China and the ornamental cultivation has a history of more than 2000 years [4]. The legume of tree peony has a star-shaped fruit. It contains dark oval seeds characterized by abundant unsaturated fatty acids (UFAs, >90%) and a high proportion of n-3 fatty acids, which has been linked with all kinds of diseases such as cancer, cardiovascular, inflammatory and autoimmune diseases, etc. [5,6]. Specially, α-linolenic acid (ALA) in tree peony oil accounts for circa 45% of the total FA content, whilst linoleic acid (LA) and oleic acid (OA) comprise ca. 26% and 21%, respectively [7-9]. With the exception of perilla seed and flaxseed oil, such high levels of n-3 FAs are uncommon in seed oils. Tree peony seeds are also abundant in phenolic and monoterpene glycosides, which are used for food and pharmaceutical purpose [10].

It is generally known that n-3 FAs are essential dietary nutrients, but cannot be synthesized by the human body independently. The recommended dietary n-6 to n-3 FA ratio is lower than 5:1 [11]. A lack of n-3 dietary intake causes the high ratio in our daily diet up to as much as 15:1 to 20:1. Due to the increasing accumulation of environmental pollutants and overharvesting, plants rich in n-3 FAs may offer a sustainable source of these essential FAs instead of fish. Currently, the most significant vegetable dietary form of n-3 FAs is ALA. However, ALA in most common edible oil, such as olive oil, corn oil, peanut oil, camellia oil, sesame oil and sunflower oil, is less than 3% [12-14]. Considerable research efforts are being put towards the exploration of new ALA-enriched resources, including the seeds of Sacha Inchi (ca. 50%), tree peony (ca. 45%), sea buckthorn (ca. 39%), cypress (ca. 35%), and cress (ca. 30%), etc. [8,15-18]. The unique health benefit of tree peony oil lies in the low n-6 to n-3 ratio (3:5), and it could be sustainable exploited as an alternative source of edible oil. Simultaneously, it is a good model to dissect metabolic pathways involved in UFA biosynthesis.

The increased ease and efficiency of RNA sequencing (RNA-Seq) tools will facilitate the study of the mechanisms underlying metabolite variation. *De novo* transcriptome sequencing has enabled the rapid identification and profiling of differentially expressed genes in flaxseed [19], castor bean [20], soybean [21], olive [22], peanut [23], sea buckthorn [24], Sacha Inchi [25], and oil palm [26]. These oilseed datasets provide large numbers of targeted gene information and can be referenced. However, due to the temporal and spatial characteristics of transcriptome, it is essential to explore the transcriptome of tree peony seeds during its development for further understanding the molecular mechanism of lipid biosynthesis. Nevertheless, most tree peony researchers committed to the molecular mechanism of flower bud development [27], endo-dormancy [28], reblooming [29], and prolonging vase life of cut flowers [30]. The physiological mechanism responsible for UFA biosynthesis remains unknown, meanwhile, the genetic control of the ALA accumulation is currently unexplored. In this study, we analyzed the FA compositions in developing tree peony seeds and generated the first tree peony seed transcriptome using high-throughput Illumina sequencing technology to uncover genes related to oil biosynthesis. In total, 175,874 contigs were obtained from six tree peony seeds transcriptome within three developing stages, and we identified most of the genes involved in fatty acid biosynthesis. The assembled, annotated transcriptome sequences and gene expression profiles establish a basis for dissecting metabolic pathways involved in UFA and TAG biosynthesis. It may provide new insights for the use of genetic engineering to increase the ALA content of traditional crops as well as its breeding.

## Results

### Lipid accumulation at different stages of seed development

**Figure 1:**
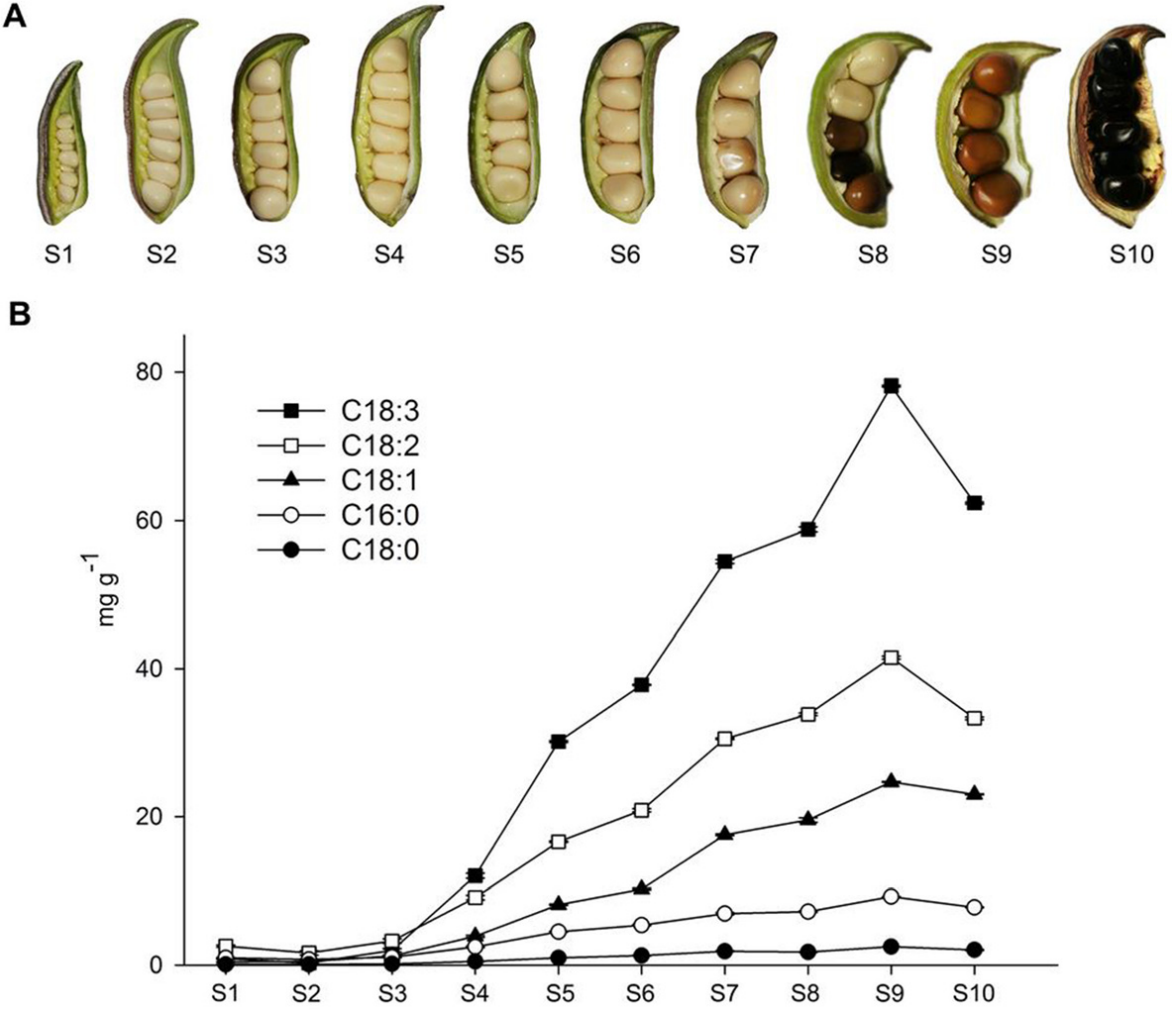
**Observation and measurement of lipids across the developmental period of tree peony seeds. (A)** The developmental progress of *P. ostii* seeds (S1-S10). Pods were harvested at 10 days after pollination (DAP, immature stage), and then every 10 days until 100 DAP (pods containing mature seeds). **(B)** The five dominant fatty acids at ten time points during tree peony seed development (mean ± SD, n = 3).

Seeds of *P. ostii* at different developmental stages were harvested and flash frozen. The methodology validated in our previous study was applied to analyze the FA composition by gas chromatography–mass spectrometry at ten developmental stages [8]. As shown in Figure 1, there were five dominant components, namely, α-linolenic acid (C18:3Δ^9c, 12c, 15c^, 49.3% of total FAs at S9), linoleic acid (C18:2Δ^9c, 12c^, 26.2%), oleic acid (C18:1Δ^9c^, 15.6%), palmitic acid (C16:0, 5.8%), and stearic acid (C18:0, 1.6%). The combined content of these five FAs always predominated across the ten developmental stages at high percentages (more than 98.5% of total FA). The other four minor FAs (<1.5%) were also detectable at trace levels including myristic acid (C14:0), *cis*-11-octadecenoic acid (C18:1Δ^11c^), eicosanoic acid (C20:0), and *cis*-11-eicosenoic acid (C20:1Δ^11c^). Although the FA compositions and contents achieved in this study were not completely identical to the results of previous studies, the identity of the dominant compounds (ALA as dominant and LA as subdominant) and the high proportion of UFAs were consistent [7,8]. Additionally, the presence of γ-linolenic acid (GLA) was reported in the seed kernel and coat of *P. rockii* [7], while it was not detected in other researches, neither in the present study. We had demonstrated that GLA did not exist in the seeds of 60 tree peony cultivars by co-elution of GLA and ALA mix [8], which was confirmed in this study due to lack of expression information of genes encoding for delta-6 desaturase through transcriptome analysis.

The accumulation of major FAs during seed development was examined, finding that FA contents varied markedly as seeds matured. Ten developmental time points can be divided into three different periods, with low level of FAs in the initial period (S1-S3), followed by a period of rapid oil accumulation (S3-S9), and a subsequent decline as seeds approaching full maturation (S9-S10) (Figure 1A). Total FAs in *P. ostii* seeds increased almost continuously throughout developmental stages and reached maxima of 158.44 mg g^−1^ at S9. While, UFAs—mainly ALA, LA, and OA—were major contributors to this notable increase (Figure 1B). FA levels of the five major compounds increased uninterrupted during maturation, reached maxima at S9 and had a slight decrease at S10 (Figure 1B). Previous reports showed that the activity and abundance of β-oxidation enzymes were increased during seed dehydration and maturity, resulting in the degradation of storage oil and a loss of lipids [31,32]. The result was very important for guiding the optimum harvest time of tree peony seeds in order to obtain maximum yield of the oil. Among all fatty acids, UFAs dominated with relatively high proportions ranging from 74.2% to 92.1% during the course of seed maturation (Additional file 1). It exhibited an extraordinary ALA content in *P. ostii*, followed by LA and OA (Additional file 1). As seeds matured, ALA content increased from an initial 0.28 mg g^−1^ to 78.18 mg g^−1^. Meanwhile, LA and OA were maintained at relatively high levels and exhibited the same tendency as ALA did during seed development (Figure 1B).

### Illumina paired-end sequencing and *de novo* assembly

In order to explore the molecular mechanism of seeds development, lipids synthesis and accumulation in tree peony, six cDNA libraries constituting two biological repeats were constructed from three stages of developing seeds (i.e., the initial stage S3, the fast oil accumulation stage S6 and the highest content stage S9) (Figure 1) and sequenced using Illumina high-throughput sequencing platform. The data sets from each stage were compared pairwise, and the correlation of duplicate samples was determined by the Pearson correlation coefficient. The high Pearson correlation coefficient, ranging from 0.929 to 0.995, indicated high reproducibility between replica samples. We obtained a total of 144 million 100-bp paired-end reads from six libraries (24 million for each), encompassing 4.8 Gb of the sequence data for each library (Table 1). After stringent quality assessment and data filtering, 23.2, 23.5, 23.4, 23.2, 23.2 and 23.2 million reads with a base quality score greater than 20 were selected and deposited in the National Center for Biotechnology Information (NCBI) Short Read Archive (accession number: SRP051810). The GC contents of the six libraries were 49.2%, 48.8% (S3), 49.9%, 49.7% (S6), 47.8% and 47.8% (S9), respectively (Table 1).

**Table 1:** **Summary of tree peony seed transcriptome data sequenced by Illumina platform**

	**S3-1**	**S3-2**	**S6-1**	**S6-2**	**S9-1**	**S9-2**
Total reads	24000000	24000000	24000000	24000000	24000000	24000000
Clean reads	23178989	23460290	23381675	23229451	23157379	23218522
Clean data (Gb)	4.64	4.70	4.68	4.65	4.63	4.64
GC percentage (%)	49.2%	48.8%	49.9%	49.7%	47.8%	47.8%

**Figure 2:**
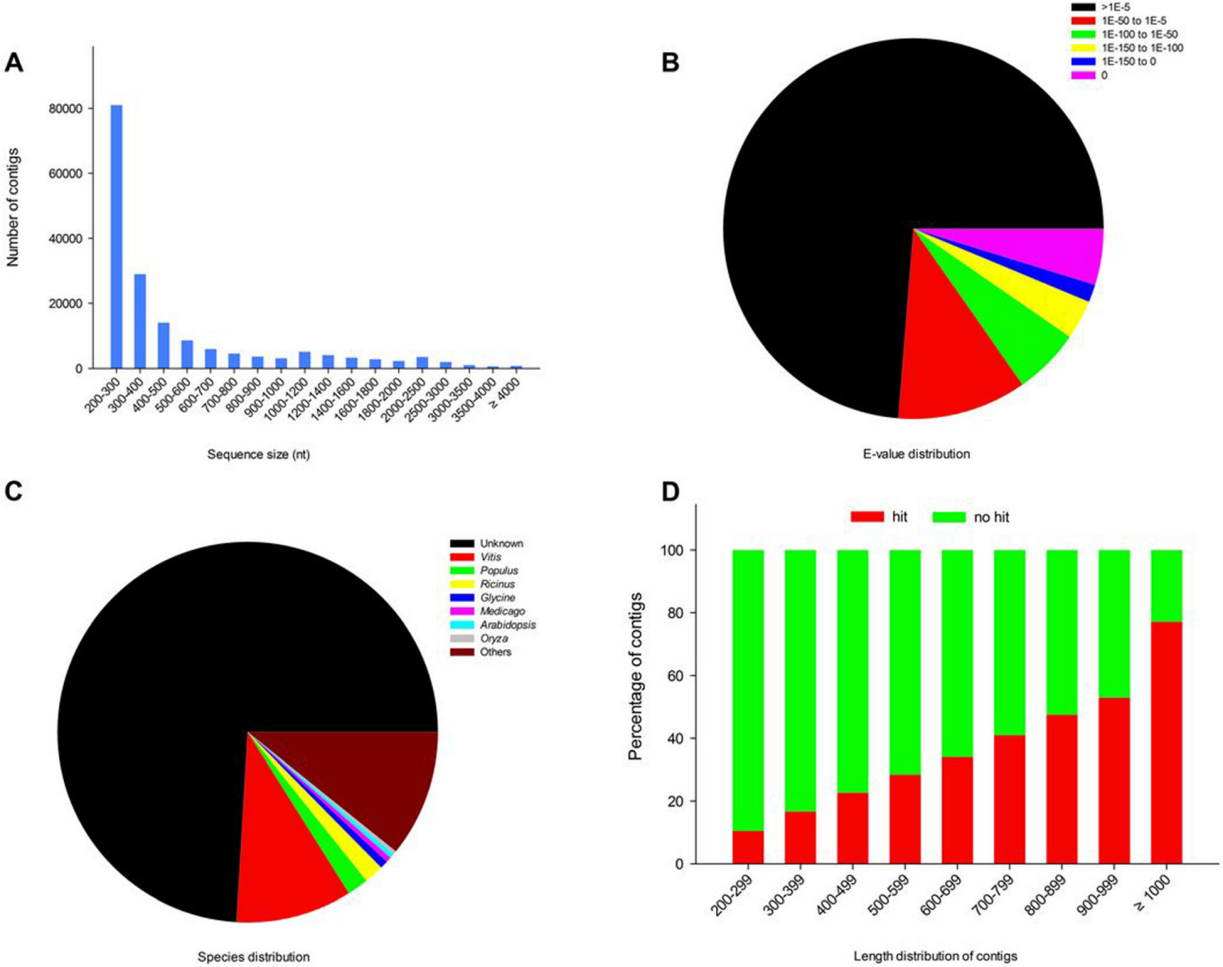
**Characteristics of Illumina reads and homology search of assembled contigs. (A)** Size distribution of tree peony Illumina reads. **(B)** E-value distribution of best BLASTX hits for each unigenes. **(C)** Species distribution of top BLAST hits of tree peony sequences with other plant species. **(D)** Length of unigenes with hits or no hits.

The *de novo* transcriptome assembly performed with Trinity with default parameters, which was developed specifically for *de novo* assembly of full-length transcripts [33]. In total, 175,874 non-redundant contigs ranging from 201 to16,860 nt long with a mean length of 581.4 nt were obtained with a total length of 97.52 Mb (Additional file 2). The size distribution of contigs showed that 25,783 contigs (14.66%) were longer than 1000 nt (Figure 2A).

### Characterization of the nonredundant unigenes of tree peony

Of the 175,874 nonredundant contigs, we tried to annotate possible open reading frames (ORFs) and their further inferred protein sequences by the program GetORF in the EMBOSS package [34]. In total, the protein coding regions of 175,874 contigs were predicted.

To annotate these genes, a homology search using the BLASTX program (E-value cut-off, 1.0E-5) revealed that 45,677 (25.97%), 12,726(7.24%), 13,405 (7.62%), 34,461 (19.59%) and 19,426 (11.04%) of the 175,874 tree peony contigs had significant matches with sequences in the NCBI nonredundant, GO, KOG, KEGG and Uniprot protein databases, respectively. These genes were tentatively annotated according to the known sequences with the best match. Altogether, 46,206 (26.27%) genes were successfully annotated based on public databases, and the low annotation rate may be attributable to the limited genomic information for tree peony. The E-value distribution of the top hits demonstrated that 15.26% of the unigenes had strong homology to previously deposited sequences (<1.0E-50), and 10.99% ranged from 1.0E-5 to 1.0E-50 (Figure 2B). Likewise, the species with the best match for each gene showed 9.87% matches with that of *Vitis*, 1.80% with popular (*Populus*), 1.66% with castor bean (*Ricinus*), and 0.68% with *Glycine max* (Figure 2C). It was in coincided with earlier studies, which fits nicely with tree peony being woody perennial [28,30]. While, 55.95% genes less than 300 nt long, could not be matched to known genes (Figure 2D), suggesting that the shorter sequences may lack a characterized protein domain or may be too short to show sequence matches, resulting in false-negative results. Due to lack of genomic and transcriptome information from seeds of tree peony in databases, other genes longer than 1 kb (4.54% of total unigenes) without hits may be considered putative novel transcribed sequences which is worthy to be studied in future.

### Functional classification of tree peony genes by Gene Ontology, Eukaryotic Orthologous Groups, and KEGG

**Figure 3:**
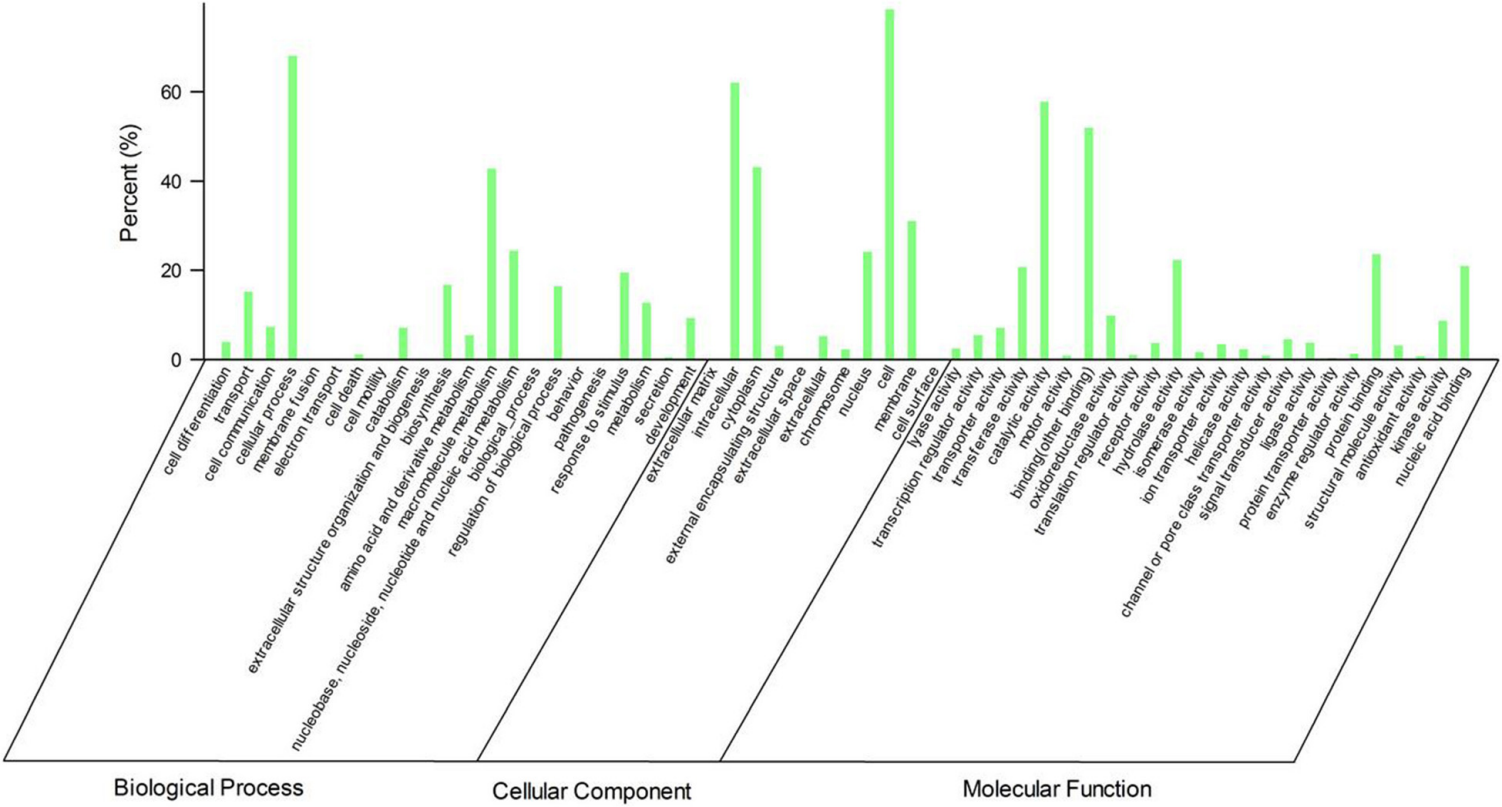
**Functional classification of Gene Ontology annotation of tree peony unigenes.** Unigenes were assigned to three categories: biological processes, cellular components, or molecular functions.

Functional annotation using gene ontology (GO) terms was carried out using the BLASTp algorithm against the Swiss Prot and TrEMBL databases by the GoPipe program according to gene2go software (E-value cut-off, 1.0E-5) [35]. Altogether, a total of 12,726 predicted proteins were categorized into 57 functional groups under three main divisions (biological processes, cellular components, and molecular functions) (Figure 3). In the biological processes category, cellular process (26.97%) and macromolecule metabolism (16.96%) were the predominant groups, followed by nucleobase, nucleoside, nucleotide and nucleic acid metabolism (9.67%), response to stimulus (7.75%), biosynthesis (6.64%), regulation of biological process (6.52%), and transport (6.01%). In the cellular components category, cell (31.38%) and intracellular (24.80%) were the most representative ones, followed by cytoplasm (17.27%), membrane (12.42%), and nucleus (9.68%). With regard to molecular function category, the predominant categories were catalytic activity (22.17%) and binding (other binding, 19.97%), followed by protein binding (9.09%), hydrolase activity (8.57%), nucleic acid binding (8.06%) and transferase activity (7.96%). The distribution of genes in different functional categories in tree peony seeds were different from transcripts of flowers for reblooming and prolonging vase life due to the spatial and temporal characteristics of transcripts [29,30].

**Figure 4:**
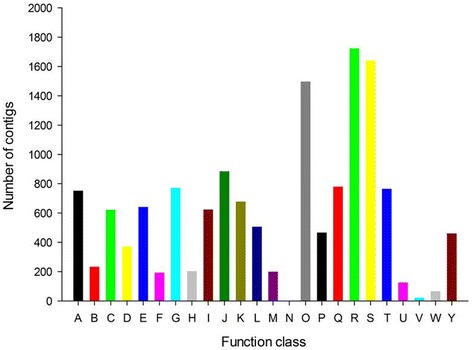
**Eukaryotic of orthologous groups (KOG) classification of assembled unigenes.** A: RNA processing and modification. B: Chromatin structure and dynamics. C: Energy production and conversion. D: Cell cycle control, cell division, chromosome partitioning. E: Amino acid transport and metabolism. F: Nucleotide transport and metabolism. G: Carbohydrate transport and metabolism. H: Coenzyme transport and metabolism. I: Lipid transport and metabolism. J: Translation, ribosomal structure and biogenesis. K: Transcription. L: Replication, recombination and repair. M: Cell wall/membrane/envelope biogenesis. N: Cell motility. O: Posttranslational modification, protein turnover and chaperones. P: Inorganic ion transport and metabolism. Q: Secondary metabolites biosynthesis, transport and catabolism. R: General function prediction only. S: Signal transduction mechanisms. T: Intracellular trafficking, secretion, and vesicular transport. U: Defense mechanisms. V: Extracellular structures. W: Nuclear structure. Y: Cytoskeleton.

To further evaluate the completeness of the transcriptome and the effectiveness of the annotation process, the annotated unigenes were compared with the eukaryotic orthologous groups (KOG) database for functional prediction and classification. In total, 14,244 annotated putative proteins were classified functionally into 24 KOG groups (Figure 4). Among the 24 categories, “signal transduction mechanisms” represented the largest group (1,641; 11.52%), followed by transcripts associated with “post-translational modification, protein turnover and chaperones” (1,498; 10.52%). The categories “cell motility” (2; 0.01%), “extracellular structures” (23; 0.16%), nuclear structure (67; 0.47%) and “defense mechanisms” (126; 0.88%) represented the smallest groups. However, categories with no concrete assignment “general function prediction only” (1,724; 12.01%) accounted for a large fraction of transcripts.

Pathway-based analysis can further our understanding of the biological functions and interactions of genes. A total of 7,065 genes were assigned to 167 pathways in the KEGG database. The most represented pathways included “ribosome” (215 unigenes), “biosynthesis of amino acids” (187 unigenes) and “carbon metabolism” (174 unigenes) (Additional file 3). Notably, some pathways were closely linked to changes in oil content and composition responsible for tree peony seed ripening, such as “fatty acid biosynthesis” (24 unigenes), “biosynthesis of unsaturated fatty acids” (22 unigenes), “α-linolenic acid metabolism” (21 unigenes), “fatty acid elongation” (18 unigenes), “glycerolipid metabolism” (39 unigenes) and “glycerophospholipid metabolism” (56 unigenes) (Additional file 3). These identified unigenes for lipids provided critical clues to identify and characterize key functional genes involved in unsaturated FA and TAG biosynthesis in tree peony seeds.

### Analysis of differentially expressed genes during tree peony seed development

**Figure 5:**
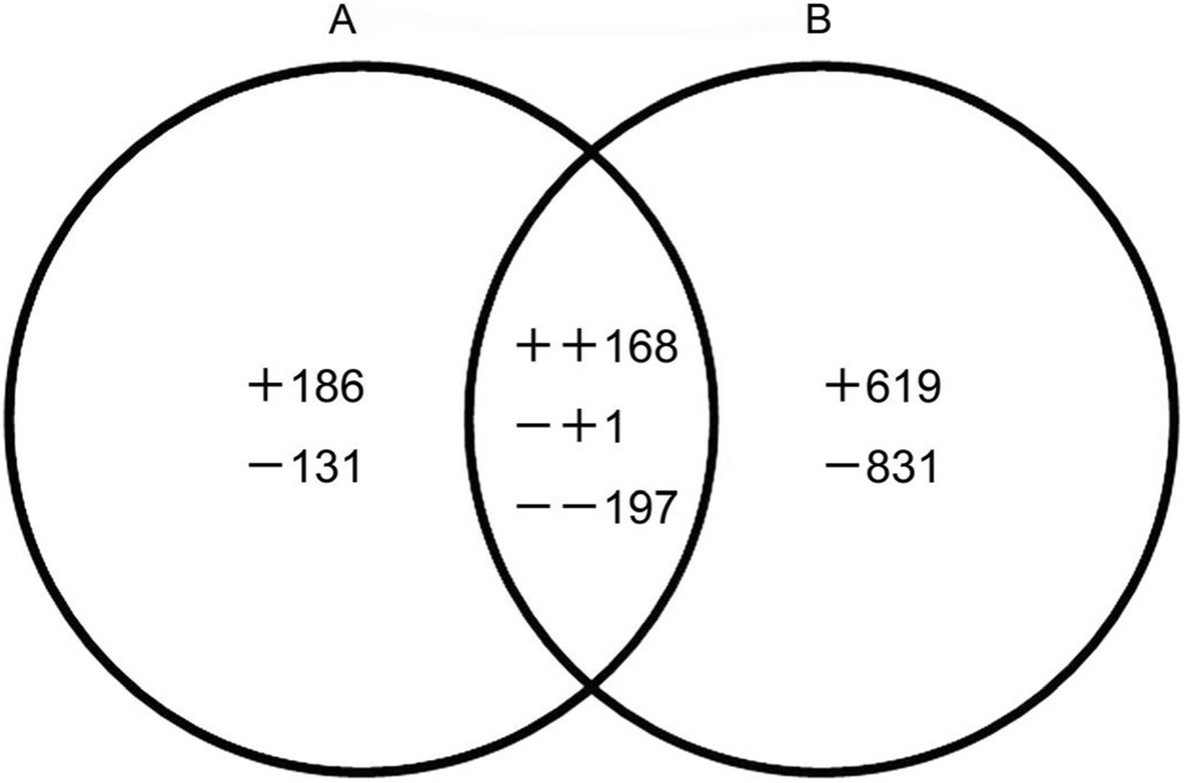
**Venn diagram of transcripts (both identified and unknown) that were up- or down-regulated between seeds of 60 DAP vs 30 DAP (A) and 90 DAP vs 30 DAP (B).** The “+” and “−” indicate up- and down-regulated transcripts, respectively. A total of 2133 transcripts were significantly (FDR < 0.05) expressed during developing seeds. 186: unique up-regulated transcripts in seeds of 60 DAP vs 30 DAP; 619: unique up-regulated transcripts in seeds of 90 DAP vs 30 DAP; 131: unique down-regulated transcripts in seeds of 60 DAP vs 30 DAP; 831: unique down-regulated transcripts in seeds of 90 DAP vs 30 DAP; 168: commonly up-regulated transcripts between seeds of 60 DAP vs 30 DAP and 90 DAP vs 30 DAP; 197: commonly down-regulated transcripts between seeds of 60 DAP vs 30 DAP and 90 DAP vs 30 DAP; 1: down-regulated in seeds of 60 DAP vs 30 DAP but up-regulated in seeds of 90 DAP vs 30 DAP.

Tissues at 30 days after pollination (DAP) were served as the control, and a total of 683 and 1816 differentially expressed unigenes were identified at 60 and 90 DAP, respectively. Among them, 366 unigenes were mutually present in both contrast groups, while 317 and 1450 were specifically expressed only in the 60 vs 30 DAP and 90 vs 30 DAP groups, respectively (Figure 5).

There were 354 and 788 up-regulated unigenes in both contrast groups, respectively, while 329 and 1028 unigenes were down-regulated. Among the up-regulated unigenes, 168 were co-expressed genes showing ≥2-fold expression difference (log_2_ ratio ≥ 1), in contrast, 186 and 619 of the up-regulated genes were specific to each respective group. Among the down-regulated unigenes, 197 were co-expressed genes showing decreases of at least 2-fold (log_2_ ratio ≤ −1), whilst, 131 and 831 of the down-regulated genes were unique to each respective group (Figure 5).

Differentially expressed genes (DEGs) provide clues about the molecular events related to seed development. Further investigation of DEGs may be warranted to determine the functional roles they played in the developmental tree peony seeds especially in the synthesis and accumulation of lipids. To evaluate the potential functions of genes that showed significant transcriptional changes between the two contrast groups, 747 differentially expressed genes with a Gene Ontology annotation were further classified into subsets. In terms of the categories, the two contrast groups showed similar patterns (Additional file 4). These genes were related to 16 biological processes, including cellular process, macromolecule metabolism, response to stimulus, and metabolism. With regard to cellular components, the analysis revealed a high percentage of transcripts in the cell, intracellular, cytoplasm, membrane, and nucleus. The unigenes were finally classified into 24 categories based on molecular function, and the two most overrepresented groups were binding (other binding, protein binding, nucleic acid binding) and catalysis (catalytic activity, transferase activity, hydrolase activity, oxidoreductase activity). To exploring the significantly enriched terms compared with the genome background, the hypergeometric test was used to map all DEGs in the GO database. The results showed that most of the characterized topological modules were assigned unambiguously to at least one cellular function, including extracellular, external encapsulating structure, metabolism, response to stimulus, and oxidoreductase activity, etc. From the large datasets, we found that down-regulated genes were more abundant than those of up-regulated genes during tree peony seed development, which was in coincidence with genes during soybean seed development [21]. KEGG functional enrichment analysis was performed to uncover their biological functions. The most represented pathways within contrast group 60 DAP vs 30 DAP included xenobiotics biodegradation and metabolism (12 enzymes), biosynthesis of other secondary metabolites (12), cell growth and death (16), metabolism of terpenoids and polyketides (8), and lipid metabolism (14). In the contrast group 90 DAP vs 30 DAP, the most represented KEGG pathways were carbohydrate metabolism (185), biosynthesis of other secondary metabolites (136), energy metabolism (64), xenobiotics biodegradation and metabolism (31), amino acid metabolism (71), lipid metabolism (52), metabolism of other amino acids (27), and metabolism of terpenoids and polyketides (23) (Additional file 5). In this study, it was worthy to be mentioned that unigenes in the pathways responsible for seed oil biosynthesis included FA biosynthesis, biosynthesis of UFAs, fatty acid elongation, glycerolipid metabolism and glycerophospholipid metabolism may provide valuable resource for the identification of unique genes involved in ALA synthesis.

### Identification and characterization of genes involved in fatty acids and triacylglycerols biosynthesis in tree peony seeds

**Figure 6:**
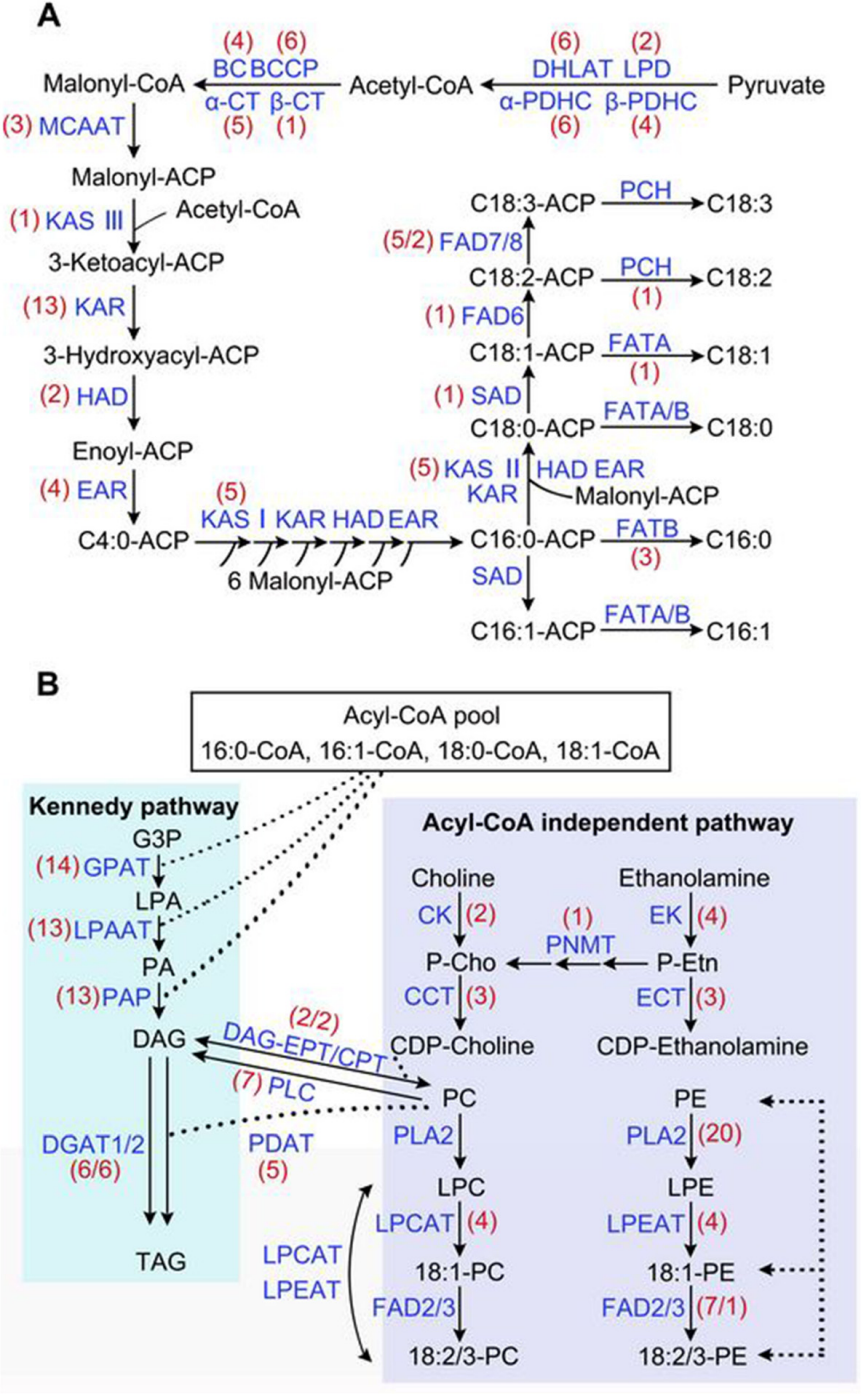
**Tree peony sequences associated with** ***de novo*** **fatty acid (A) and triacylglycerols (B) biosynthetic pathways.** Lipid substrates are abbreviated: C16:0, palmitic acid; C16:1, hexadecenoic acid; C18:0, stearic acid; C18:1, oleic acid; C18:2, linoleic acid; C18:3, linolenic acid. Enzyme/protein abbreviations are: α-PDHC, pyruvate dehydrogenase alpha subunit; β-PDHC, pyruvate dehydrogenase beta subunit; DHLAT, dihydrolipoamide acetyltransferase; LPD, dihydrolipoamide dehydrogenase; α-CT, carboxyl transferase alpha subunit; β-CT, carboxyl transferase beta subunit; BC, biotin carboxylase; BCCP, biotin carboxyl carrier protein; MCAAT, malonyl-CoA ACP transacylase; ACP, acyl carrier protein; KAS I, II, III, ketoacyl-ACP synthase I, II, III; KAR, ketoacyl-ACP reductase; HAD, hydroxyacyl-ACP dehydrase; EAR, enoyl-ACP reductase; SAD, stearoyl-ACP desaturase; FAD6, oleate desaturase (chloroplast); FAD8, linoleate desaturase (chloroplast); FAD2, oleate desaturase (endoplasmic reticulum); FAD3, linoleate desaturase (microsomal); FATA/B, acyl-ACP thioesterase A/B; PCH, palmitoyl-CoA hydrolase; GPAT, glycerol-3-phosphate acyltransferase; LPAAT, 1-acylglycerol-3-phosphate acyltransferase; PAP, phosphatidic acid phosphatase; DGAT1/2, acyl-CoA: diacylglycerolacyltransferase 1/2; PLA2/C, phospholipase A2/C; CK, choline kinase; CCT, choline-phosphate cytidylyltransferase; LPCAT, 1-acylglycerol-3-phosphocholine acyltransferase; EK, ethanolamine kinase; ECT, ethanolamine-phosphate cytidylyltransferase; LPEAT, 1-acylglycerol-3-phosphoethanolamine acyltransferase; PDAT, phospholipid:diacylglycerolacyltransferase; DAG-EPT, DAG-ethanolaminephosphotransferase.

Most enzymes involved in the lipid biosynthesis were identified in the tree peony seed transcriptome. In total, 388 unigenes implicated in FA biosynthesis and TAG assemblage were identified (Additional file 6). Among the differentially expressed genes, more were down-regulated, and some showed a differential expression pattern between three contrast groups. Pathway-based analysis helps to clarify the biological functions of genes. Thereof, 106 genes were involved in the initiation and acyl chain elongation steps of *de novo* FA biosynthesis (Additional file 6, Initiation and Elongation). Most of these genes had at least one isoform significantly down-regulated in S9 compared with S3, suggesting their functional involvement in the beginning of FA biosynthesis. The acetyl-CoA carboxylase (ACCase) catalyzed the first reaction to generate an intermediate malonyl-CoA, which is a regulatory enzyme that controls, at least in part, the rate of fatty acid synthesis (Figure 6A). It was reported that plastidic ACCase was the enzymatic target of feedback inhibition in tobacco suspension cells and *Brassica napus* embryo-derived cells [36,37]. Subsequently, repeated condensations of malonyl-ACP with a growing acyl-ACP chain were primed by fatty acid synthase (FAS) subunits, consecutively adding two carbon units to form 16:0-ACP (Figure 6A). Unigenes comp38788 and comp79316 encoding KAS II (elongation of palmitoyl-ACP to stearoyl-ACP) were highly expressed at S3, which was consistent with the high proportion of 18-carbon fatty acids in tree peony seeds [38]. The FAS generally terminated at saturated 16-carbon fatty acids in the plastid, which is catalyzed by palmitoyl-ACP thioesterase [39]. Unigenes comp52856, comp76036_c0_seq2, and comp76036_c0_seq5 encoding FATA and FATB were down-regulated more than 4-fold compared with that of S9 to S3. In addition, 21 unigenes were identified to encode long-chain acyl-CoA synthetases (LACS), and four unigenes encoded acyl-CoA binding proteins (ACBP), generating the ER acyl-CoA pool (Figure 6B).

In oilseeds, the major fate of newly synthesized FAs is exported from plastids as acyl-CoA to enter the glycerolipid synthesis [40]. Triacylglycerols are the main components of storage lipids in oil seed plants, with the major constituents being 16- and 18-carbon FAs. Generally, TAG can be synthesized through the sequential acyl-CoA dependent acylation of a glycerol backbone (known as the Kennedy pathway), in which 62 unigenes were identified in tree peony seeds (Figure 6B) [41]. TAG can also be formed in plants via two different acyl-CoA independent pathways, catalyzed by phospholipid:diacylglycerolacyltransferase (PDAT) and diacylglyceroltransacylase (DGTA), in which DGTA was absent in tree peony seeds [42]. Unigenes encoding PDAT had low transcript levels, implying that this route might not be a major pathway in tree peony seeds (Figure 6B and Additional file 6). In contrast, unigenes relevant to acyl editing, PE-DAG and PC-DAG interconversion were relatively active, which was believed to facilitate the incorporation of UFAs into TAG in tree peony seeds [43,44].

Among the 103 unigenes encoding phospholipases, 18 unigenes had relatively higher transcript levels (Additional file 6, Phospholipases). These unigenes may be related to the formation of membrane lipids and cell division. While, lipases that arose in developing seeds of castor bean was indicated to take part in remodeling of TAGs after synthesis [20,45]. Totally, 19 unigenes encoding triacylglycerol lipase were identified from our libraries. The function of these genes in tree peony developing seeds needed to be further studied. In the mature oilseed, TAGs are stored in the form of small subcellular spherical oil bodies [46]. Five unigenes encoding oil-body oleosins were identified with high expression level in developing seeds (Additional file 6, Oil accumulation). Unigenes comp55793, comp81207, comp68377, comp92379, and comp84664 were of 2-fold to 16-fold up-regulation, suggesting their active involvement in oil accumulation in tree peony seeds. The above identified specific genes would provide clues to understand the molecular mechanism on oil accumulation of tree peony seeds.

Most FAs produced in the plastid were not immediately available for TAG biosynthesis before desaturation. In tree peony seeds, 18 unigenes encoding fatty acid desaturase (FAD) were detected, including one unigenes for stearoyl-ACP desaturase (SAD), nine unigenes for oleate desaturase (eight for FAD2 and one for FAD6) and eight unigenes for omega-3 fatty acid desaturase (one for FAD3, five for FAD7 and two for FAD8) (Additional file 6, Desaturation). There were no related unigenes encoding FAD4 and FAD5 detected in tree peony seeds. Fatty acid desaturase is responsible for sequential modification of OA to LA and ALA. FAD2 and FAD6 remove two hydrogen atoms from OA to form LA, while FAD3, FAD7 and FAD8 catalyze the conversion from LA to ALA [40]. However, most of them exhibited low expression levels across seed development, and only three unigenes were differentially expressed. Thereinto, the abundant transcripts of comp79170 and comp84636 separately encoding SAD and FAD2 in S3 and S6 were consistent with the relatively high levels of oleic acid and its downstream products. The expression of comp77478 encoding FAD8 was up-regulated 2.69-fold in S6 and then down-regulated 9.65-fold in S9, which indicated its correspondent function in ALA accumulation. Further cloning and functional analyses of the three genes would probably reveal the molecular mechanisms underlying UFAs biosynthesis in tree peony seeds.

### Expression analysis of candidate DEGs related to seed development

We analyzed transcript abundance by qRT-PCR in tree peony seeds at three different developmental stages described in Figure 1 (S3, S6 and S9). According to the genes annotated in KEGG and the results of lipid biosynthesis obtained in many plants [24,25], ten DEGs were selected for expression analysis (Additional file 7). A reliable R^2^ correlation coefficient of 0.74 was obtained between qRT-PCR and RNA-Seq results by linear regression analyses (Additional file 8), confirming the validity of the transcriptome results.

**Figure 7:**
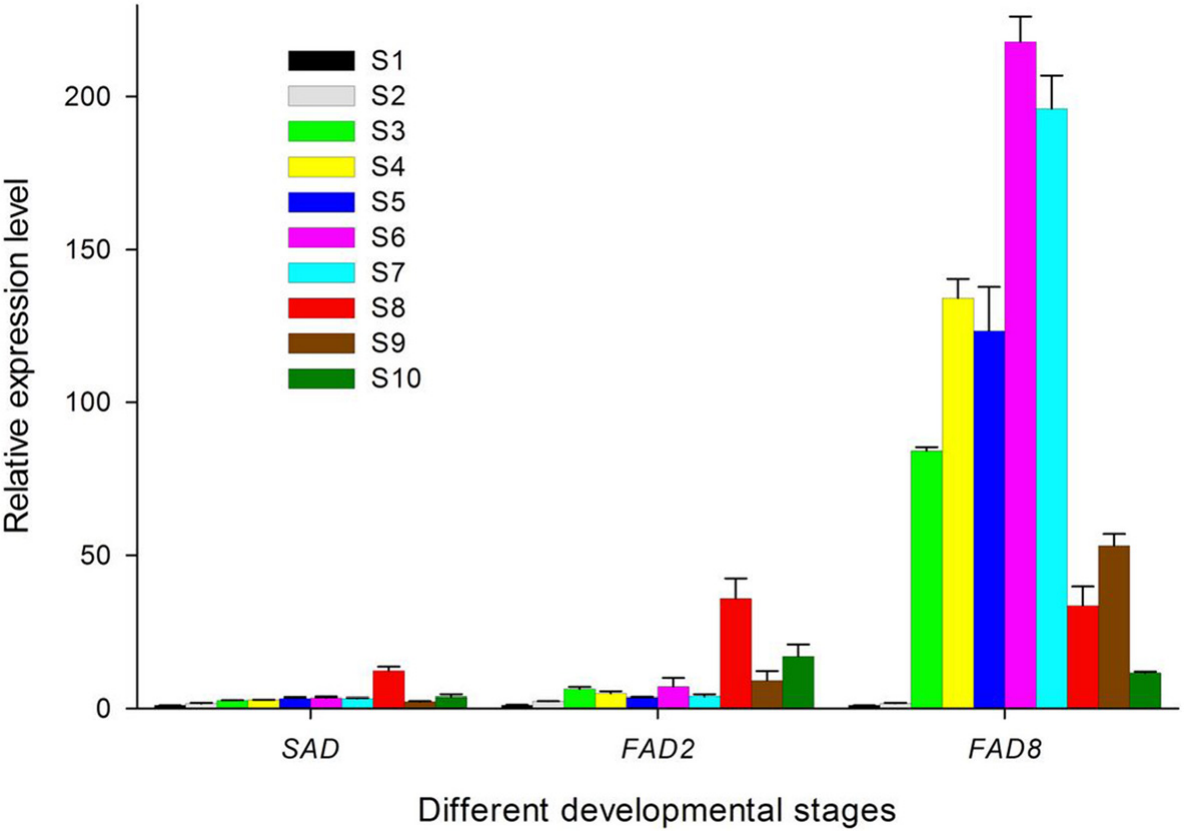
**RT-PCR analysis of genes involved in fatty acid desaturation in** ***P. ostii*** **seed at different developmental stages.** Relative expression values, normalized to *ubiquitin*, were shown as 2^−ΔΔCt^ relative to 10 DAP. Error bars represent the SD of three biological replicas with three technical replicas each.

To see if any correlations existed between UFA accumulation in developing seeds and expression patterns of desaturation genes, we analyzed transcript abundance of three related genes (*SAD*, *FAD2* and *FAD8*) by qRT-PCR from ten different developmental stages described in Figure 1. The three genes catalyzed the sequential desaturation of stearic acid at Δ9, Δ12 and Δ15 position, generating oleic acid, linoleic acid and α-linolenic acid, respectively. The results indicated that they were expressed across the seed development period, but the levels of expression varied considerably at different developmental stages (Figure 7). Genes exhibited relatively low transcript abundance during initial development (S1-S2), and then their expression increased gradually (S3-S8) and dropped thereafter (S9-S10). The significantly high-level expression of *FAD8* than *SAD* and *FAD2* after S3 was in agreement with the higher ALA content than OA and LA. Specifically, expression of the desaturases *SAD* and *FAD2* peaked in the S8, consistent with the maximum OA and LA contents observed in the seed oil at S9. The reduced expression of both genes in later stages of seed development indicated a reduced accumulation of oleic acid and linoleic acid at S10 (Figure 1 Figure 7). The expression of *FAD8* was characterized by a bell-shape transcript curve, with low levels of expression at the initial stage followed by a substantial increase during the rapid phase of oil accumulation and a subsequent decline toward seed maturation. The similar gene profiles in *Brassica napus*, Arabidopsis and sea buckthorn were observed [24,45,47]. The high levels of *FAD8* transcripts during critical developmental period (S3-S7) coincided with the high number of reads obtained for this gene by Illumina sequencing (Figure 7).

## Discussion

ALA exerts a wide range of biological activities (e.g., as a nootropic) and prophylactic effects (including anti-hyperlipidemic, anti-inflammatory, anti-thrombotic, and anti-hypertensive) [48-50]. Due to the high value and growing demand for ALA, exploiting oleaginous resources and dissecting the mechanism of oil biosynthesis and UFA accumulation have been extensively studied [22,51,52]. Recent years, tree peony is expectedly gaining popularity as a resource in food and nutraceutical industries. Compared with traditional oil crops—soybean, rapeseed, peanut, corn, sunflower, etc., tree peony oil, with its high ALA level (ca. 45%) together with a near 3:5 ratio of n-6 to n-3 fatty acids, represents a very balanced source of polyunsaturated fatty acids for human health and nutrition. The medical and nutritional applications of tree peony oil warrant further exploration and it can be developed as important nutritional supplements for specific populations, weanling infants, preschool children, and pregnant and lactating mothers, etc. [5,12]. Furthermore, the statistics indicate that the global oil consumption from oilseed crops was approximately 165.3 million tons in 2013 [53]. Vegetable oil consumption is expected to double by 2040 [54]. Therefore, considerable research efforts related to the exploration of new oleaginous resources and oil biosynthesis in seeds are urgently needed, and it is believed to be a promising avenue for the next decade.

Many plants deposit TAG in seeds and fruits as the major form of storage lipid, which is stored in densely packed oil bodies [55]. The acyl-CoA-dependent pathway catalyzed by diacylglycerol acyltransferase (DGAT) is considered the major pathway for TAG assembly in plants [40]. TAG can also be formed by two acyl-CoA independent acyltransferase (PDAT and DGTA) [42]. The expression levels of genes encoding DGAT and PDAT were relatively low, while DGTA was absent in tree peony seed transcript in this study. Nevertheless, it was surprising that genes related to oilbody formation were still largely expressed. The characterized phenomenon has remained elusive since the temporal patterns of gene expression for the enzymes relevant to TAG assembly are very different during seed development. So far, there is a fundamental lack of clarity about the regulation of gene expression and enzyme activity for these later steps in oil biosynthesis [56,57].

The diversity of UFA composition in TAG could be enriched by phosphatidylcholine (PC) or phosphatidylethanolamine (PE) acyl editing. PC-DAG and PE-DAG interconversion allowed acyl-CoA newly exported from the plastid to enter PC or PE for FA modification, while desaturated FAs depart for TAG. Alternatively, the acyl in DAG was converted to PC or PE by DAG-cholinephosphotransferase (DAG-CPT) and DAG-ethanolaminephosphotransferase (DAG-EPT). The reversibility of this reaction allows the entire DAG portion of PC or PE, including any modified FAs, to participate in TAG synthesis [58]. The vast majority of FAs (>90%) within the seed flux through PC before incorporation into TAG, suggesting that PC is a central intermediate in the flux of FAs or DAG, or both substrates into TAG [59]. However, genes encoding DAG-CPT were weakly expressed in our libraries, but it did not mean the inactivity of PC-DAG interconversion. Previous researches showed that *Saccharomyces cerevisiae* ethanolaminephosphotransferase activity was capable of efficiently utilizing CDP-ethanolamine, CDP-monomethylethanolamine, CDP-dimethylethanolamine, and CDP-choline *in vitro*. This broad substrate specificity implied that *EPT1* gene product might synthesize both PE and PC *in vivo* [44,46]. Additionally, PC also can be synthesized from PE by two rounds of methylation [40].

Desaturation is the key step that resulted in the desirable n-3 and n-6 fatty acids. *Arabidopsis thaliana* mutants have provided the evidence that there are seven genetic loci, *fad2, fad3, fad4, fad5, fad6, fad7* and *fad8*, in which *fad3*, *fad7* and *fad8* encoded omega-3 fatty acid desaturase [40]. Generally, the contents of UFAs were consistent with expression levels of genes for desaturases. Genes in tree peony seed corresponded to those from the seeds of flax, Sacha Inchi and sea buckthorn, suggesting that the regulatory pathways involved in the FAs biosynthesis are conserved. *FAD3* genes would be specifically expressed in perilla and Sacha Inchi seeds, in which ALA accumulations were more than 50% of the total fatty acid contents [25,60]. In the sea buckthorn transcriptome, *FAD2*, *FAD3*, *FAD6*, *FAD7* and *FAD8* were expressed at similar levels, which was consistent with the 1:1 ratio of n-6:n-3 fatty acids [24]. These plant resources enriched in ALA differed in the mechanism of fatty acid desaturation, signifying their unique metabolic pathways in the process of evolution [19,24,25]. *FAD2* and *FAD8* were highly expressed in tree peony seeds, indicating that *FAD2* and *FAD8* might play a vital role in the formation of polyunsaturated fatty acids. The verification of selected genes with qRT-PCR confirmed the changes, in which the relative expression level of *FAD8* was much higher than *FAD2*. We could deduce that the high content of ALA in tree peony seeds was attributed to the activity and abundance of FAD8 enzymes. Further studies related to expression, substrate specificity or regulation of activity of enzymes related to fatty acids desaturation in tree peony seeds will shed light on the understanding of its special synthetic pathway, and simultaneously lay a foundation for the artificial regulation and molecular breeding.

## Conclusions

In conclusion, lipid accumulation across developing tree peony seeds was monitored. Our results indicate that tree peony oil can benefit human health in the long run owing to its high levels of α-linolenic acid (49.3%) and a low ratio of n-6/n-3 ration (3:5). Additionally, we report here the first transcript dataset derived by using high-throughput sequencing technology (Illumina) for tree peony seed. Transcriptome analyses from three developmental stages of tree peony seeds resulted in 175,874 contigs. Expression of 683 and 1816 unigenes differed at least 2-fold in the 60 and 90 days after pollination compared to the initial stage, respectively. Focusing on lipid genes, we identified 388 unigenes associated with the *de novo* FA biosynthesis, the formation of UFAs, TAG assembly and oleosin formation from tree peony seeds. These unigenes provide a comprehensive molecular biology background for researches on tree peony seed development, particularly with respect to the process of oil accumulation. In particular, those candidate genes (such as *SAD*, *FAD2*, *FAD8*) associated with UFA biosynthesis would provide critical clues to reveal the molecular mechanisms underlying the high levels of α-linolenic acid biosynthesis in tree peony seeds.

## Methods

### Plant material

Seeds of *P. ostii* were collected in 2012 at Beijing Botanical Garden, Institute of Botany, Chinese Academy of Sciences (Lat. 39°48’ N, 116°28’ E, Alt. 76 m), China. It had been introduced to the garden and grown in the same eco-environmental and cultivation conditions for ten years. We observed the seed development process from pollination till maturation in May-August, 2012. Pods were hand-collected at intervals of ten days, from the beginning of podding until full maturity (Figure 1), covering a total range of 100 d, and the oil content of developing seeds were measured as described in our previous study [8]. For the transcriptome sampling, seeds at three developing stages (30, 60, 90 DAP) were collected from the same individual, and pods with the same developing stage from two trees comprised of two replicates. The collected samples were flash frozen in liquid nitrogen and stored at −80°C until further use.

### Total RNA extraction, cDNA library construction, and high throughput sequencing

Total RNA was extracted from the seeds collected at 30, 60 and 90 DAP, respectively, using a TIANGEN RNA Prep Pure Plant kit (Tiangen Biotech Co. Ltd, Beijing, China), and purified with the Dynabeads® Oligo (dT)25 kit (Life, USA). RNA integrity was evaluated with a 2.0% agarose gel stained with Goldview. The yield and quality of the RNA samples were determined using a NanoDrop®ND-1000 spectrophotometer. The cDNA library was constructed by using a NEBNext® Ultra RNA Library Prep Kit, the quality of retrieved cDNA was checked using the Agilent 2100 Bioanalyzer (RNA Nano Chip, Agilent), and it was sequenced using an Illumina HiSeq™ 2000 paired-end sequencing system.

### Sequences analysis and assembly

Sequencing-received raw image data were transformed by base calling into sequence data. Low quality reads and reads with adaptors or N% more than 5% were removed before data analysis. Clean reads from six libraries were assembled using the program Trinity [33], which is an efficient method for *de novo* assembly of full-length transcripts. Trinity combined three independent software modules: Inchworm, Chrysalis and Butterfly. Firstly, reads with overlapping sequences were merged to form contigs using Inchworm, which often generate full-length transcripts for a dominant isoform. Secondly, Chrysalis clustered the contigs into clusters and constructed complete de Bruijn graphs for each cluster, each graph reflecting the full transcriptional complexity for a given gene. Finally, the de Bruijn graphs were then processed and paired-end matched to form longer sequences using Butterfly, resolving alternatively spliced isoforms and transcripts derived from paralogous genes. Gene prediction was determined by EMBOSS package. Contigs were aligned by BLASTX (E-value <1e − 5) to the NCBI nonredundant, GO (http://www.geneontology.org/), Swiss-Prot (http://www.ebi.ac.uk/uniprot/), KOG (http://www.ncbi.nlm.nih.gov/COG/), and KEGG (Kyoto Encyclopedia of Genes and Genomes; http://www.genome.jp/kegg/) protein databases to annotate the functions. We used the GoPipe to determine Gene Ontology annotation of contigs [35]. KEGG Orthology annotations of the unigenes were conducted using BlastX algorithm against KEGG database.

### Differential expression of unigenes

The DESeq package in the R software environment was used to analyze differential gene expression between developing stages. The DESeq models count data using a negative binomial distribution, with variance and mean linked by local regression [61]. P-values were adjusted for multiple testing using the Benjamini-Hochberg false discovery rate (FDR) correction (p < 0.05). P-value <0.05 and absolute value of the log_2_ ratio >1 were used as the threshold to determine significant differences in gene expression.

### Gene Ontology and KEGG Orthology enrichment analyses for differentially expressed unigenes

Gene Ontology and KEGG Orthology enrichment analyses of the differentially expressed genes were then carried out. Enriched p-values were calculated according to the hypergeometric test [62]. All p-values were adjusted with the Bonferroni correction. We selected the corrected p-value of 0.05 as the threshold to determine significant enrichment of the gene sets. For KEGG Orthology enrichment analysis, we used the false discovery rate 0.05 as the threshold to determine significant enrichment of the gene sets.

### Quantitative RT-PCR analysis

Total RNA was isolated from frozen seeds of S1-S10. cDNA synthesis was prepared with 1 μg total RNA using PrimeScript® RT reagent Kit With gDNA Eraser (TaKaRa, Japan) according to manufacturer’s instructions. Q-PCR was performed with SYBR® Premix Ex TaqTM (Perfect Real Time) (TaKaRa, Japan). Gene transcript levels were quantified by real-time PCR with the Roche LightCycler 480 instrument (Roche, Mannheim, Germany). The following standard thermal profile was used for all PCR experiments: 95°C for 10 min; 40 cycles of 95°C for 15 s and 60°C for 1 min. Fluorescence signals were captured at the end of each cycle, and the melting curve analysis was performed from 65°C to 95°C. The *ubiquitin* gene was used as internal control in this study [63]. The amplification system (e.g., primer and template concentrations) was properly optimized, and the efficiency was close to 1. Relative expression levels of target genes were calculated by the 2^-ΔΔCt^ comparative threshold cycle (Ct) method [64]. All gene-specific primers in this paper were showed in Additional file 7.

## Additional files

Additional file 1:
**Fatty acid contents in developing seeds of** ***P. ostii*** **(mg g** ^**−1**^ **FW).**
Additional file 2:
**FASTA sequences of contigs over all stages of development.**
Additional file 3:
**KEGG categories of nonredundant unigenes in tree peony.**
Additional file 4:
**Gene Ontology categories of unigenes with significant transcriptional changes during different stages of seed development.**
Additional file 5:
**KEGG Orthology enrichment analysis of unigenes with significant transcriptional changes during seed development at different stages.**
Additional file 6:
**Enzymes/proteins related to lipid accumulation in tree peony seeds.**
Additional file 7:
**Gene-specific primers sequence for detection by qRT-PCR.**
Additional file 8:
**Linear regression analysis between qRT-PCR and RNA-Seq results for ten genes.**

## Abbreviations

ALA: α-linolenic acid
FA: Fatty acid
UFA: Unsaturated fatty acid
LA: Linoleic acid
OA: Oleic acid
PA: Palmitic acid
SA: Stearic acid
GLA: γ-linolenic acid
TAG: Triacylglycerol
ACP: Acyl carrier protein
ACCase: Acetyl-COA carboxylase
KAS: Ketoacyl-ACP synthase
FATA/B: Thioesterase A/B
DAG: Diacylglycerol
PC: Phosphatidylcholine
PE: Phosphatidylethanolamine
DAP: Days after pollination
ER: Endoplasmic reticulum
GO: Gene ontology
KOG: Eukaryotic orthologous groups
DEG: Differentially expressed genes
SAD: Stearoyl-ACP desaturase
FAS: Fatty acid synthase
FAD: Fatty acid desaturase
GPAT: Glycerol-3-phosphate acyltransferase
LPAAT: Lysophosphatidic acid acyltransferase
DGAT: Diacylglycerolacyltransferase
EST: Expressed sequence tag
