# Supplementary material for: Fatty acid composition of developing tree peony (Paeonia section Moutan DC.) seeds and transcriptome analysis during seed development
Source: BMC Genomics. 2015 Mar 18;16(1):208. doi: 10.1186/s12864-015-1429-0 (PMC4404109; doi:10.1186/s12864-015-1429-0)
Supplement: Additional file 1: — Fatty acid contents in developing seeds of P. ostii (mg g −1 FW). [file 12864_2015_1429_MOESM1_ESM.docx]

**Additional file 1 Fatty acid contents in developing seeds of *P. ostii* (mg g^-1^ FW)**

|  | S1 | S2 | S3 | S4 | S5 | S6 | S7 | S8 | S9 | S10 |
| --- | --- | --- | --- | --- | --- | --- | --- | --- | --- | --- |
| C14:0 |  |  |  |  |  |  | 0.05±0.001 | 0.05±0.001 | 0.05±0.003 | 0.05±0.001 |
| C16:0 | 0.97±0.06 | 0.75±0.08 | 1.07±0.10 | 2.45±0.09 | 4.51±0.05 | 5.38±0.09 | 6.95±0.15 | 7.22±0.11 | 9.24±0.02 | 7.79±0.04 |
| C18:0 | 0.09±0.02 | 0.09±0.02 | 0.16±0.03 | 0.49±0.02 | 0.98±0.02 | 1.29±0.07 | 1.87±0.06 | 1.76±0.04 | 2.48±0.01 | 2.05±0.06 |
| C18:1△^9c^ | 0.41±0.04 | 0.5±0.03 | 1.20±0.13 | 3.89±0.13 | 8.14±0.10 | 10.24±0.12 | 17.54±0.12 | 19.56±0.30 | 24.71±0.07 | 23.02±0.08 |
| C18:1△^11c^ |  |  |  | 0.08±0.001 | 0.16±0.001 | 0.31±0.01 | 0.42±0.001 | 0.50±0.01 | 0.53±0.004 | 0.46±0.003 |
| C18:2△^9c,12c^ | 2.56±0.15 | 1.64±0.29 | 3.24±0.31 | 9.1±0.31 | 16.62±0.10 | 20.86±0.22 | 30.54±0.11 | 33.85±0.18 | 41.51±0.21 | 33.30±0.19 |
| C20:0 |  |  |  |  |  |  |  | 0.64±0.48 | 1.09±0.01 | 1.04±0.02 |
| C18:3△^9c,12c,15c^ | 0.85±0.1 | 0.28±0.01 | 1.99±0.22 | 12.03±0.34 | 30.15±0.13 | 37.82±0.08 | 54.44±0.26 | 58.8±0.35 | 78.18±0.12 | 62.36±0.13 |
| C20:1△^11c^ |  |  |  |  |  |  |  |  | 0.65±0.003 | 0.33±0.002 |
| Total FA | 4.88 | 3.26 | 7.66 | 28.04 | 60.56 | 75.9 | 111.81 | 122.38 | 158.44 | 130.4 |
| n-3% | 17.4 | 8.6 | 26.0 | 42.9 | 49.8 | 49.8 | 48.7 | 48.0 | 49.3 | 47.8 |
| n-6% | 52.5 | 50.3 | 42.3 | 32.5 | 27.4 | 27.5 | 27.3 | 27.7 | 26.2 | 25.5 |
| UFA% | 78.3 | 74.2 | 83.9 | 89.5 | 90.9 | 91.2 | 92.1 | 92.1 | 91.9 | 91.6 |
