# Supplementary material for: Fatty acid composition of developing tree peony (Paeonia section Moutan DC.) seeds and transcriptome analysis during seed development
Source: BMC Genomics. 2015 Mar 18;16(1):208. doi: 10.1186/s12864-015-1429-0 (PMC4404109; doi:10.1186/s12864-015-1429-0)
Supplement: Additional file 3: — KEGG categories of nonredundant unigenes in tree peony. [file 12864_2015_1429_MOESM3_ESM.docx]

**Additional file 3 KEGG categories of nonredundant unigenes in tree peony**

|  | **Pathway** | **Count** | **Pathway ID** |
| --- | --- | --- | --- |
| 1 | Ribosome | 215 | ko03010 |
| 2 | Biosynthesis of amino acids | 187 | ko01230 |
| 3 | Carbon metabolism | 174 | ko01200 |
| 4 | Spliceosome | 172 | ko03040 |
| 5 | RNA transport | 169 | ko03013 |
| 6 | Protein processing in endoplasmic reticulum | 159 | ko04141 |
| 7 | Purine metabolism | 146 | ko00230 |
| 8 | Oxidative phosphorylation | 121 | ko00190 |
| 9 | Pyrimidine metabolism | 117 | ko00240 |
| 10 | Plant hormone signal transduction | 111 | ko04075 |
| 11 | mRNA surveillance pathway | 106 | ko03015 |
| 12 | Ubiquitin mediated proteolysis | 104 | ko04120 |
| 13 | Starch and sucrose metabolism | 98 | ko00500 |
| 14 | Ribosome biogenesis in eukaryotes | 92 | ko03008 |
| 15 | RNA degradation | 89 | ko03018 |
| 16 | Cell cycle | 88 | ko04110 |
| 17 | Amino sugar and nucleotide sugar metabolism | 80 | ko00520 |
| 18 | Glycolysis / Gluconeogenesis | 80 | ko00010 |
| 19 | Endocytosis | 77 | ko04144 |
| 20 | Plant-pathogen interaction | 74 | ko04626 |
| 21 | Aminoacyl-tRNA biosynthesis | 70 | ko00970 |
| 22 | Pyruvate metabolism | 65 | ko00620 |
| 23 | Peroxisome | 64 | ko04146 |
| 24 | Glutathione metabolism | 63 | ko00480 |
| 25 | Arginine and proline metabolism | 62 | ko00330 |
| 26 | Nucleotide excision repair | 61 | ko03420 |
| 27 | Oocyte meiosis | 60 | ko04114 |
| 28 | Cysteine and methionine metabolism | 58 | ko00270 |
| 29 | Proteasome | 56 | ko03050 |
| 30 | Glycerophospholipid metabolism | 56 | ko00564 |
| 31 | Glycine, serine and threonine metabolism | 54 | ko00260 |
| 32 | Phagosome | 53 | ko04145 |
| 33 | Basal transcription factors | 53 | ko03022 |
| 34 | Carbon fixation in photosynthetic organisms | 53 | ko00710 |
| 35 | DNA replication | 52 | ko03030 |
| 36 | Lysosome | 51 | ko04142 |
| 37 | Glyoxylate and dicarboxylate metabolism | 49 | ko00630 |
| 38 | 2-Oxocarboxylic acid metabolism | 48 | ko01210 |
| 39 | N-Glycan biosynthesis | 48 | ko00510 |
| 40 | Regulation of actin cytoskeleton | 46 | ko04810 |
| 41 | Homologous recombination | 46 | ko03440 |
| 42 | Phenylpropanoid biosynthesis | 46 | ko00940 |
|  |  |  |  |
| 43 | Photosynthesis | 46 | ko00195 |
| 44 | RNA polymerase | 45 | ko03020 |
| 45 | Terpenoid backbone biosynthesis | 44 | ko00900 |
| 46 | Inositol phosphate metabolism | 44 | ko00562 |
| 47 | Pentose phosphate pathway | 44 | ko00030 |
| 48 | Citrate cycle (TCA cycle) | 43 | ko00020 |
| 49 | Phosphatidylinositol signaling system | 41 | ko04070 |
| 50 | Alanine, aspartate and glutamate metabolism | 41 | ko00250 |
| 51 | Base excision repair | 40 | ko03410 |
| 52 | Galactose metabolism | 40 | ko00052 |
| 53 | Porphyrin and chlorophyll metabolism | 39 | ko00860 |
| 54 | Glycerolipid metabolism | 39 | ko00561 |
| 55 | Fructose and mannose metabolism | 39 | ko00051 |
| 56 | Mismatch repair | 38 | ko03430 |
| 57 | Phenylalanine, tyrosine and tryptophan biosynthesis | 38 | ko00400 |
| 58 | Phenylalanine metabolism | 38 | ko00360 |
| 59 | Valine, leucine and isoleucine degradation | 38 | ko00280 |
| 60 | Protein export | 37 | ko03060 |
| 61 | SNARE interactions in vesicular transport | 36 | ko04130 |
| 62 | Fc gamma R-mediated phagocytosis | 35 | ko04666 |
| 63 | Glycosylphosphatidylinositol(GPI)-anchor biosynthesis | 33 | ko00563 |
| 64 | Propanoate metabolism | 31 | ko00640 |
| 65 | Fatty acid metabolism | 31 | ko00071 |
| 66 | Pentose and glucuronate interconversions | 29 | ko00040 |
| 67 | Nitrogen metabolism | 28 | ko00910 |
| 68 | Various types of N-glycan biosynthesis | 28 | ko00513 |
| 69 | beta-Alanine metabolism | 28 | ko00410 |
| 70 | Tyrosine metabolism | 28 | ko00350 |
| 71 | Ascorbate and aldarate metabolism | 28 | ko00053 |
| 72 | Cytosolic DNA-sensing pathway | 27 | ko04623 |
| 73 | MAPK signaling pathway | 26 | ko04010 |
| 74 | Pantothenate and CoA biosynthesis | 26 | ko00770 |
| 75 | Ubiquinone and other terpenoid-quinone biosynthesis | 26 | ko00130 |
| 76 | Cyanoamino acid metabolism | 25 | ko00460 |
| 77 | Focal adhesion | 24 | ko04510 |
| 78 | One carbon pool by folate | 24 | ko00670 |
| 79 | Fatty acid biosynthesis | 24 | ko00061 |
| 80 | Circadian rhythm - plant | 23 | ko04712 |
| 81 | Tight junction | 23 | ko04530 |
| 82 | Axon guidance | 22 | ko04360 |
| 83 | Biosynthesis of unsaturated fatty acids | 22 | ko01040 |
| 84 | Sulfur metabolism | 22 | ko00920 |
| 85 | Stilbenoid, diarylheptanoid and gingerol biosynthesis | 21 | ko00945 |
| 86 | Sphingolipid metabolism | 21 | ko00600 |
| 87 | alpha-Linolenic acid metabolism | 21 | ko00592 |
| 88 | Lysine degradation | 21 | ko00310 |
| 89 | Butanoate metabolism | 20 | ko00650 |
| 90 | TGF-beta signaling pathway | 19 | ko04350 |
| 91 | Metabolism of xenobiotics by cytochrome P450 | 19 | ko00980 |
| 92 | Carotenoid biosynthesis | 19 | ko00906 |
| 93 | Limonene and pinene degradation | 18 | ko00903 |
| 94 | Aminobenzoate degradation | 18 | ko00627 |
| 95 | Arachidonic acid metabolism | 18 | ko00590 |
| 96 | Ether lipid metabolism | 18 | ko00565 |
| 97 | Selenocompound metabolism | 18 | ko00450 |
| 98 | Fatty acid elongation | 18 | ko00062 |
| 99 | Natural killer cell mediated cytotoxicity | 17 | ko04650 |
| 100 | Adherens junction | 17 | ko04520 |
| 101 | Chemokine signaling pathway | 17 | ko04062 |
| 102 | Calcium signaling pathway | 17 | ko04020 |
| 103 | Regulation of autophagy | 16 | ko04140 |
| 104 | ABC transporters | 16 | ko02010 |
| 105 | Folate biosynthesis | 16 | ko00790 |
| 106 | Polycyclic aromatic hydrocarbon degradation | 16 | ko00624 |
| 107 | Other glycan degradation | 16 | ko00511 |
| 108 | Valine, leucine and isoleucine biosynthesis | 16 | ko00290 |
| 109 | Steroid biosynthesis | 16 | ko00100 |
| 110 | Endocrine and other factor-regulated calcium reabsorption | 15 | ko04961 |
| 111 | Flavonoid biosynthesis | 15 | ko00941 |
| 112 | Biotin metabolism | 15 | ko00780 |
| 113 | Riboflavin metabolism | 15 | ko00740 |
| 114 | Tryptophan metabolism | 15 | ko00380 |
| 115 | Bisphenol degradation | 15 | ko00363 |
| 116 | Histidine metabolism | 15 | ko00340 |
| 117 | Melanogenesis | 14 | ko04916 |
| 118 | NOD-like receptor signaling pathway | 14 | ko04621 |
| 119 | Gap junction | 14 | ko04540 |
| 120 | Tropane, piperidine and pyridine alkaloid biosynthesis | 14 | ko00960 |
| 121 | Sulfur relay system | 13 | ko04122 |
| 122 | Two-component system | 13 | ko02020 |
| 123 | Nicotinate and nicotinamide metabolism | 13 | ko00760 |
| 124 | Thiamine metabolism | 13 | ko00730 |
| 125 | Photosynthesis - antenna proteins | 13 | ko00196 |
| 126 | Notch signaling pathway | 12 | ko04330 |
| 127 | Diterpenoid biosynthesis | 12 | ko00904 |
| 128 | Circadian entrainment | 11 | ko04713 |
| 129 | Vitamin B6 metabolism | 11 | ko00750 |
| 130 | Linoleic acid metabolism | 11 | ko00591 |
| 131 | Lipopolysaccharide biosynthesis | 11 | ko00540 |
| 132 | Lysine biosynthesis | 11 | ko00300 |
| 133 | Apoptosis | 10 | ko04210 |
| 134 | Circadian rhythm | 9 | ko04710 |
| 135 | Isoquinoline alkaloid biosynthesis | 9 | ko00950 |
| 136 | Sesquiterpenoid and triterpenoid biosynthesis | 9 | ko00909 |
| 137 | Non-homologous end-joining | 8 | ko03450 |
| 138 | Zeatin biosynthesis | 8 | ko00908 |
| 139 | Retinol metabolism | 8 | ko00830 |
| 140 | Glycosaminoglycan degradation | 8 | ko00531 |
| 141 | Taurine and hypotaurine metabolism | 8 | ko00430 |
| 142 | Degradation of aromatic compounds | 7 | ko01220 |
| 143 | Glycosphingolipid biosynthesis - ganglio series | 7 | ko00604 |
| 144 | Cutin, suberine and wax biosynthesis | 7 | ko00073 |
| 145 | Aldosterone-regulated sodium reabsorption | 6 | ko04960 |
| 146 | Naphthalene degradation | 6 | ko00626 |
| 147 | Glycosphingolipid biosynthesis - globo series | 6 | ko00603 |
| 148 | Brassinosteroid biosynthesis | 5 | ko00905 |
| 149 | Lipoic acid metabolism | 5 | ko00785 |
| 150 | Styrene degradation | 5 | ko00643 |
| 151 | Phosphonate and phosphinate metabolism | 5 | ko00440 |
| 152 | Synthesis and degradation of ketone bodies | 5 | ko00072 |
| 153 | C5-Branched dibasic acid metabolism | 4 | ko00660 |
| 154 | Benzoate degradation | 4 | ko00362 |
| 155 | Flavone and flavonol biosynthesis | 3 | ko00944 |
| 156 | Monoterpenoid biosynthesis | 3 | ko00902 |
| 157 | Steroid hormone biosynthesis | 3 | ko00140 |
| 158 | Glucosinolate biosynthesis | 2 | ko00966 |
| 159 | Betalain biosynthesis | 2 | ko00965 |
| 160 | Polyketide sugar unit biosynthesis | 2 | ko00523 |
| 161 | Other types of O-glycan biosynthesis | 2 | ko00514 |
| 162 | D-Glutamine and D-glutamate metabolism | 2 | ko00471 |
| 163 | Geraniol degradation | 2 | ko00281 |
| 164 | Caffeine metabolism | 2 | ko00232 |
| 165 | Anthocyanin biosynthesis | 1 | ko00942 |
| 166 | Peptidoglycan biosynthesis | 1 | ko00550 |
| 167 | Mucin type O-glycan biosynthesis | 1 | ko00512 |
