# Supplementary material for: Fatty acid composition of developing tree peony (Paeonia section Moutan DC.) seeds and transcriptome analysis during seed development
Source: BMC Genomics. 2015 Mar 18;16(1):208. doi: 10.1186/s12864-015-1429-0 (PMC4404109; doi:10.1186/s12864-015-1429-0)

**Additional file 4 Gene Ontology categories of unigenes with significant transcriptional changes during different stages of seed development.**

**S6/S3**


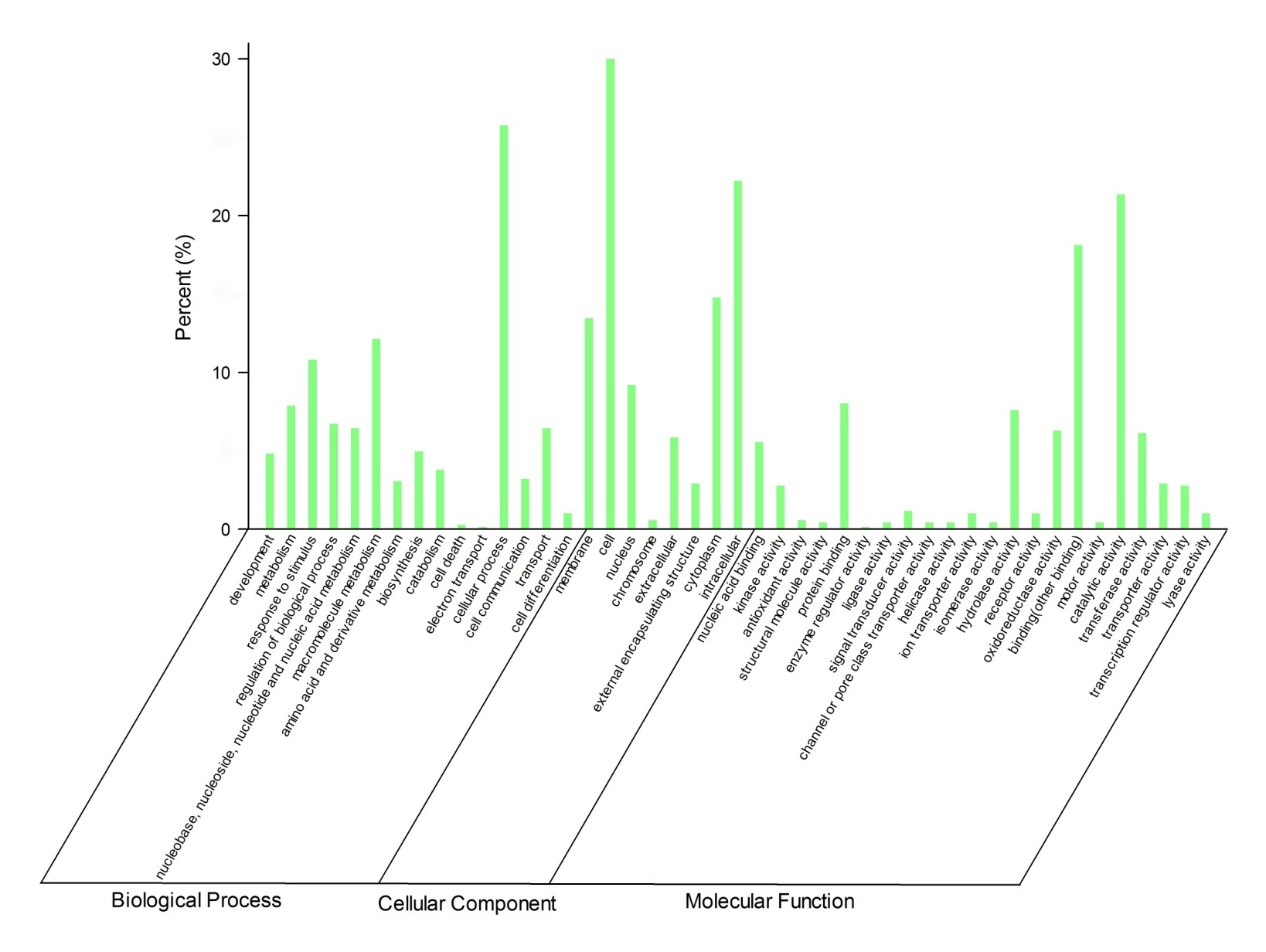


**S9/S3**


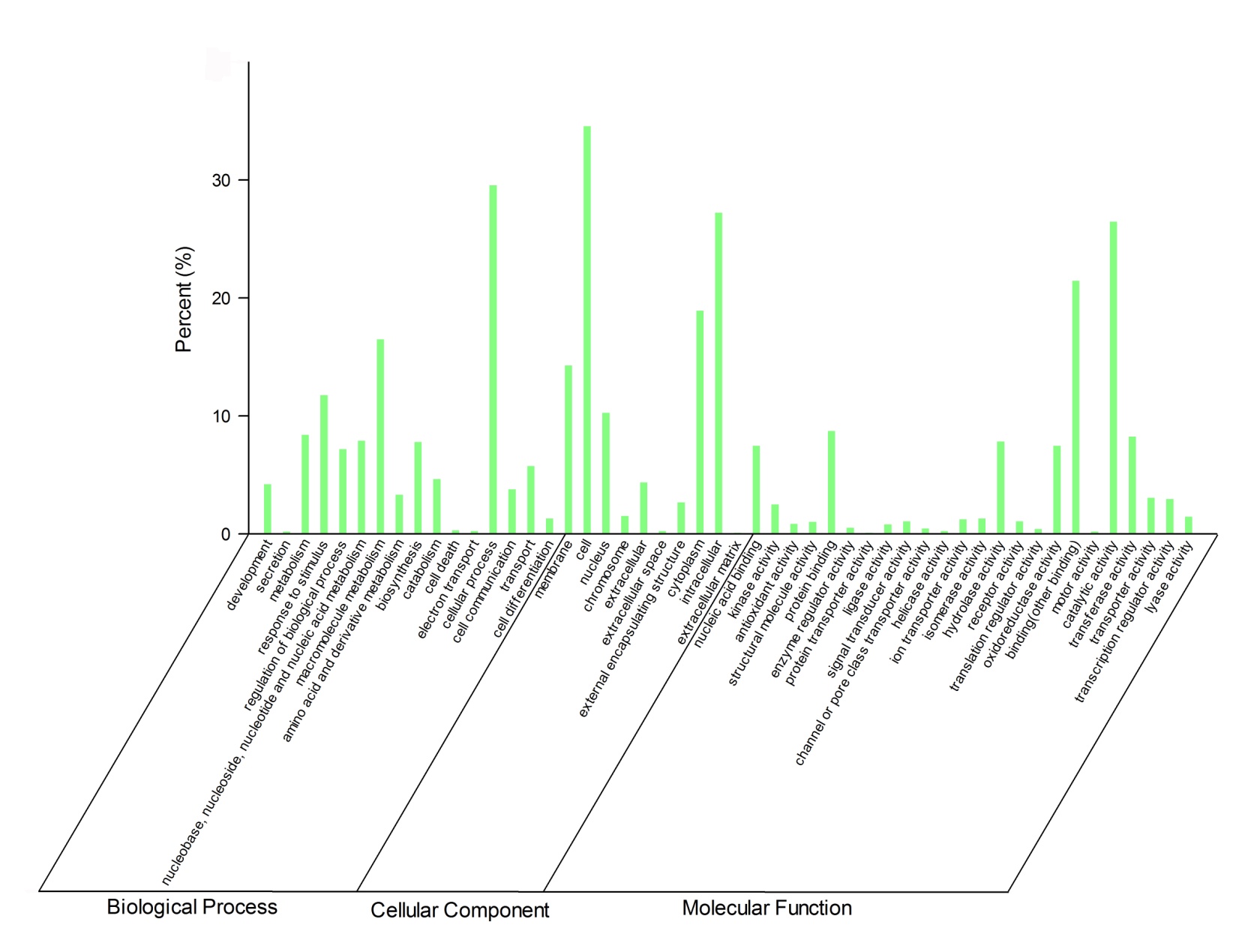

Supplement: Additional file 4: — Gene Ontology categories of unigenes with significant transcriptional changes during different stages of seed development. [file 12864_2015_1429_MOESM4_ESM.docx]
