# Supplementary material for: Fatty acid composition of developing tree peony (Paeonia section Moutan DC.) seeds and transcriptome analysis during seed development
Source: BMC Genomics. 2015 Mar 18;16(1):208. doi: 10.1186/s12864-015-1429-0 (PMC4404109; doi:10.1186/s12864-015-1429-0)
Supplement: Additional file 5: — KEGG Orthology enrichment analysis of unigenes with significant transcriptional changes during seed development at different stages. [file 12864_2015_1429_MOESM5_ESM.docx]

**Additional file 5 KEGG Orthology enrichment analysis of unigenes with significant transcriptional changes during different stages of seed development.**

**S6/S3**

| KEGG Pathway | DEGs | P_value | q_value (BH_adjust) |
| --- | --- | --- | --- |
| Xenobiotics biodegradation and metabolism | 12 | 1.16E-05 | 0.000265 |
| Biosynthesis of other secondary metabolites | 12 | 1.36E-05 | 0.000265 |
| Cell growth and death | 16 | 0.001929 | 0.025074 |
| Metabolism of terpenoids and polyketides | 8 | 0.003452 | 0.033657 |
| Lipid metabolism | 14 | 0.00545 | 0.04251 |

**S9/S3**

| KEGG Pathway | DEGs | P_value | q_value (BH_adjust) |
| --- | --- | --- | --- |
| Carbohydrate metabolism | 185 | 7.43E-45 | 2.90E-43 |
| Biosynthesis of other secondary metabolites | 45 | 4.73E-14 | 6.14E-13 |
| Energy metabolism | 64 | 1.56E-06 | 1.52E-05 |
| Xenobiotics biodegradation and metabolism | 31 | 4.05E-06 | 3.16E-05 |
| Amino acid metabolism | 71 | 2.07E-05 | 0.000134 |
| Lipid metabolism | 52 | 0.000128 | 0.000712 |
| Metabolism of other amino acids | 27 | 0.001274 | 0.00621 |
| Metabolism of terpenoids and polyketides | 23 | 0.003951 | 0.017119 |
