# Supplementary material for: Fatty acid composition of developing tree peony (Paeonia section Moutan DC.) seeds and transcriptome analysis during seed development
Source: BMC Genomics. 2015 Mar 18;16(1):208. doi: 10.1186/s12864-015-1429-0 (PMC4404109; doi:10.1186/s12864-015-1429-0)
Supplement: Additional file 7: — Gene-specific primers sequence for detection by qRT-PCR. [file 12864_2015_1429_MOESM7_ESM.docx]

Additional file 7: Gene-speciﬁc primers sequence for detection by qRT-PCR.

| Gene | Forward primer (5'-3') | Reverse primer (5'-3') |
| --- | --- | --- |
| *β-PDHC* | AGGCTGGCTTTACAGGGATT | GGGAATGTTGAGCACCAACT |
| *MCAT* | TGACGACGCTTTGTTTTCTG | CCATTTGTGCAAACATCCAG |
| *KAS II* | ACTCAACCCTCAATGCCAAG | TCGAGTTGGGAATTTGGAAG |
| *EAR* | ACTCAACCCTCAATGCCAAG | ATGTTGATTCCGCTCTCACC |
| *SAD* | GCGTCATGAGACTGCCTACA | TCGCTGAGCTACAGACGAGA |
| *FAD2* | TCACGCTTGGTGAAGTCAAG | GTAGATGGGCCAAGCAACAT |
| *FATA* | CTTGGAAGTTTGACGGAGGA | AAGGTGCAATTTCCTCATGG |
| *PLA1* | TGAAAGTTGAGTCGGGGTTC | TCCGCCATTACGTTTAGACC |
| *FAD8* | TCTTCCCTCAAATCCCACAC | GAGCTCATGGTCGGTCTTGT |
| *OBO* | GGGATCCACAGTTGCTTGTT | GGCCGGAGAGGACTAAGAGT |
| *Ubiquitin* | GACCTATACCAAGCCGAAG | CGTTCCAGCACCACAATC |
