# Supplementary figures and images for: Fatty acid composition of developing tree peony (Paeonia section Moutan DC.) seeds and transcriptome analysis during seed development
Source: BMC Genomics. 2015 Mar 18;16(1):208. doi: 10.1186/s12864-015-1429-0 (PMC4404109; doi:10.1186/s12864-015-1429-0)

Additional file 8: Linear regression analysis between qRT-PCR and RNA-Seq results for ten genes.


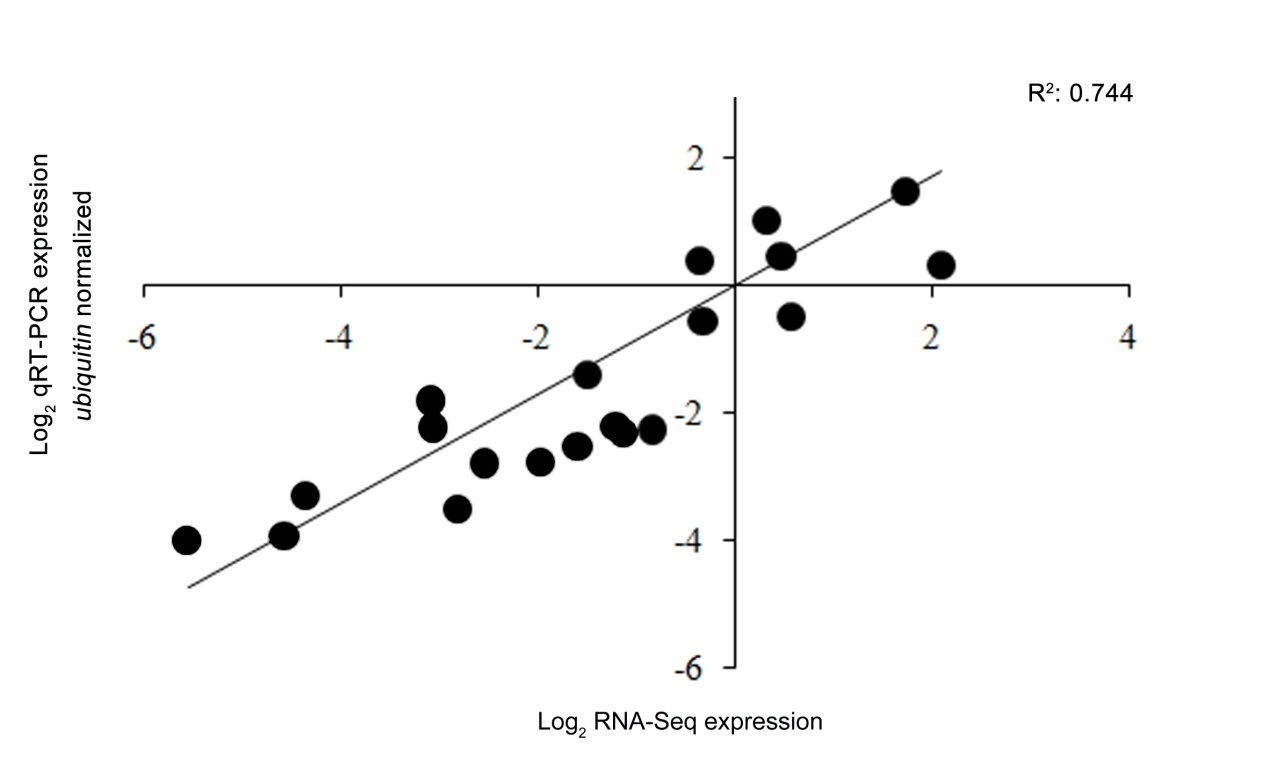

Supplement: Additional file 8: — Linear regression analysis between qRT-PCR and RNA-Seq results for ten genes. [file 12864_2015_1429_MOESM8_ESM.docx]
